# Feeding on Host Plants with Different Concentrations and Structures of Pyrrolizidine Alkaloids Impacts the Chemical-Defense Effectiveness of a Specialist Herbivore

**DOI:** 10.1371/journal.pone.0141480

**Published:** 2015-10-30

**Authors:** Carlos H. Z. Martins, Beatriz P. Cunha, Vera N. Solferini, José R. Trigo

**Affiliations:** 1 Laboratório de Ecologia Química, Departamento de Biologia Animal, Instituto de Biologia, UNICAMP, Caixa Postal 6109, 13083–970, Campinas, São Paulo, Brazil; 2 Programa de Pós-Graduação em Biologia Funcional e Molecular, Instituto de Biologia, UNICAMP, Caixa Postal 6109, 13084–970, Campinas, São Paulo, Brazil; 3 Departamento de Genética e Evolução, Instituto de Biologia, Unicamp, Caixa Postal 6109, 13083–970, Campinas, São Paulo, Brazil; University of Natural Resources and Life Sciences, AUSTRIA

## Abstract

Sequestration of chemical defenses from host plants is a strategy widely used by herbivorous insects to avoid predation. Larvae of the arctiine moth *Utetheisa ornatrix* feeding on unripe seeds and leaves of many species of Crotalaria (Leguminosae) sequester *N*-oxides of pyrrolizidine alkaloids (PAs) from these host plants, and transfer them to adults through the pupal stage. PAs confer protection against predation on all life stages of *U*. *ornatrix*. As *U*. *ornatrix* also uses other *Crotalaria* species as host plants, we evaluated whether the PA chemical defense against predation is independent of host plant use. We fed larvae from hatching to pupation with either leaves or seeds of one of eight *Crotalaria* species (*C*. *incana*, *C*. *juncea*, *C*. *micans*, *C*. *ochroleuca*, *C*. *pallida*, *C*. *paulina*, *C*. *spectabilis*, and *C*. *vitellina*), and tested if adults were preyed upon or released by the orb-weaving spider *Nephila clavipes*. We found that the protection against the spider was more effective in adults whose larvae fed on seeds, which had a higher PA concentration than leaves. The exceptions were adults from larvae fed on *C*. *paulina*, *C*. *spectabilis* and *C*. *vitellina* leaves, which showed high PA concentrations. With respect to the PA profile, we describe for the first time insect-PAs in *U*. *ornatrix*. These PAs, biosynthesized from the necine base retronecine of plant origin, or monocrotaline- and senecionine-type PAs sequestered from host plants, were equally active in moth chemical defense, in a dose-dependent manner. These results are also partially explained by host plant phylogeny, since PAs of the host plants do have a phylogenetic signal (clades with high and low PA concentrations in leaves) which is reflected in the adult defense.

## Introduction

Defenses evolved by animals to avoid predation are ubiquitous in nature, and different defensive strategies have evolved in response to different life styles. The myriad defensive strategies include avoiding detection, preventing attack, and deceiving predators [1]. Some herbivorous insects feeding on chemically protected host plants are able to overcome these plant defenses by sequestering plant secondary compounds, and using them for their own defense against predator attack [2–4]. Sequestration may have evolved independently in different taxa of herbivorous insects. It predominates in Coleoptera and Lepidoptera, but also occurs frequently in the Heteroptera, Hymenoptera, Orthoptera and Sternorrhyncha [4]. The sequestered defensive compounds comprise a vast array of natural products of different biosynthetic pathways, such as cardenolides, cyanogenic and iridoid glycosides, aristolochic acids, glucosinolates and pyrrolizidine alkaloids. These substances are effective against a variety of predators, ranging from invertebrates including spiders and ants, to vertebrates including birds and mammals [2–5].

Generally, the effectiveness of chemical defenses against predation is dose- and/or structure-dependent [3]. High concentrations of sequestered compounds in the herbivorous prey are more effective against predators, and their effectiveness is usually related to the concentration of these compounds in the host plants. Different structures of the same class of compounds may also show different activities against predation. For instance, a palatability spectrum of the monarch and queen butterflies, *Danaus plexippus* and *D*. *gilippus*, was found to be associated with the amount of cardenolides sequestered from different host plants [6,7]. This pattern has been also observed in the adults of the nymphalid butterfly *Euphydryas phaeton*, which acquired defensive iridoid glycosides as larvae from two different host plants [8,9]; and in the adults of the heliconiine butterfly *Heliconius erato*, whose larvae feed on four species of *Passiflora* [10]. Additionally, Silva and Trigo [11] demonstrated that pyrrolizidine alkaloids have a positive dose-dependent activity in the protection of insects against the orb-weaving spider *Nephila clavipes* (Nephilidae). In the same study, they showed that different PA structures had different antipredation activities.

Among the defensive compounds sequestered by herbivorous insects, the role of pyrrolizidine alkaloids (PAs) has been well documented [12]. These compounds are produced by plants in several families (e.g. Asteraceae, Boraginaceae and Leguminosae), and are sequestered by specialist grasshoppers, hemipterans, beetles, moths and butterflies [5,13,14], where they show defensive and sexual communication functions [14]. PAs in specialized insects are always present in *N*-oxide form [15]. Many arctiine moths convert PAs sequestered from their larval host plants into “insect-PAs” in which the acid components of the alkaloids are replaced by small, branched aliphatic 2-hydroxy acids of insect origin [16]. These PAs of insect origin are precursors of the male sex pheromone in these moths [16].

The arctiine rattlebox moth *Utetheisa ornatrix* is one of the most-studied species with respect to its ecological dependence on PAs [5,14,17–19]. *U*. *ornatrix* occurs in the Neotropics and warm Nearctic regions [20], where it feeds on many species of *Crotalaria* legumes [21]. The genus *Crotalaria* has a Pantropical distribution, and some members have colonized the warmer parts of the Nearctic region [22]. The larvae of *U*. *ornatrix* sequester these alkaloids, and pass them to pupae and adults. During mating, males transfer PAs to females, which transfer them to the eggs. Therefore, all life stages of *U*. *ornatrix* are protected by PAs against predators [14]. Eisner [23] first demonstrated that the unpalatability of *U*. *ornatrix* adults is due to PAs in their tissues, and that the alkaloid originates from the larval host plant, *Crotalaria pallida* (formerly *C*. *mucronata*). Eisner found that adults of *U*. *ornatrix* were protected against the orb-weaving spider *Nephila clavipes*, as well as from other spiders, and birds. Additional evidence of the defensive role of PAs in *U*. *ornatrix* came from bioassays, with PAs topically applied on palatable organisms or by offering diets with or without PAs to PA-specialist insects, and testing these organisms against various predators [5].

Although it is suggested that the unpalatability of *U*. *ornatrix* may be closely related to the amount of PAs in their host-plant tissues, few studies have demonstrated this relationship. Eisner et al. [14,23–25] tested the adults, with and without PAs, against spiders and birds, but no dose-activity bioassay was carried out. Ferro et al. [26] found that the differences in adult palatability increased when their larvae fed on unripe seeds and leaves of *C*. *pallida*. The leaves had a lower PA content than unripe seeds, and consequently the larvae fed on leaves were more consumed by *N*. *clavipes* than those fed on unripe seeds. Likewise, Hristov and Conner [27] also showed that *U*. *ornatrix* fed on leaves of *C*. *spectabilis* were more predated by the bat *Eptesicus fuscus* compared to moths fed on seeds; moths raised in a diet free of PAs were palatable to the bats.

An important issue that remains unclear is the role of PA structure in the chemical defense of *U*. *ornatrix*. Do different structures confer different levels of defense? Eisner et al. [23–25] raised *U*. *ornatrix* on its usaramine PA-containing host plant (*C*. *pallida*) and observed that the moth was rejected by the spiders *N*. *clavipes* and *Lycosa ceratiola*, while moths raised on a diet free of PAs were preyed upon. Similar results were found for *L*. *ceratiola*, which released *U*. *ornatrix* raised on a diet supplied with PA monocrotaline, the main PA of *C*. *spectabilis* [24,25]. In two other studies on chemical defense of *U*. *ornatrix* adults, larvae were also raised on *C*. *pallida* or *C*. *spectabilis*, and therefore the PAs involved in the defense were respectively, usaramine and monocrotaline [26,27]. However, larvae of *U*. *ornatrix* can feed on many *Crotalaria* species [21,28], with different PA concentrations and profiles [29]. The finding that *C*. *pallida* and *C*. *spectabilis* conferred similar levels of defense in spite of containing different PAs led us to hypothesize that the structure is unimportant in determining palatability. Our predictions were: (1) adults from larvae that fed on plants or plant parts with high PA contents would be better defended than those that fed on plants or plant parts with low PA contents, and (2) PA structure would play no role in the moth chemical defenses.

Another point that has not been examined in studies of chemical defenses of herbivores sequestered from their host plants is the role of host plant phylogeny. It would be expected that phylogenetically related host plants produce similar compounds available for sequestration by insects. Therefore we cannot consider each host plant species as independent for comparative analysis (e.g. [30]). If phylogenetic inertia is strong, the potential adaptations that related species may evolve will be similarly constrained, with the effect that species cannot be regarded as independent of each other [31]. Consequently, it was necessary to take into account the host plant phylogeny, in order to compare the sequestered chemical defenses in an herbivorous insect. To our knowledge, this approach has never been taken in published studies. For example, even for the monarch butterfly, a well-studied model of sequestered chemical compounds, the connection between insect defenses and host plant phylogenetic relationships has never been explored, although the phylogenetic trends related to chemical defenses are well known in *Asclepias* [32,33]. Therefore, we explored the question of whether *Crotalaria* phylogeny directs any trend for the chemical defenses of *U*. *ornatrix*. We hypothesized that moths that fed on phylogenetically related host plants would show similar defensive patterns.

To address our hypothesis and predictions, we fed larvae on leaves or seeds of eight different *Crotalaria* species found in the Neotropics, which were native, non-native or of uncertain origin, with different PA concentrations and profiles [29]. The adults that emerged from larvae fed the different diets were offered to the spider *N*. *clavipes* in a predation bioassay. We analyzed the PA concentrations and profiles for *U*. *ornatrix* adults and *Crotalaria* species, and correlated the PA concentrations of adults with the PA contents of the plant parts they fed on as larvae. We also correlated the PA concentration and profile between the moths and the spider response in the predator bioassay. This correlation will or will not support our first prediction. Since we found different classes of PAs in *U*. *ornatrix*, we also bioassayed the three most common classes against the spider to test our second prediction. Finally, we mapped the PA profiles and concentrations in *Crotalaria* species, the PA concentrations in the moth, and the defensive response of *N*. *clavipes* against an independent phylogenetic hypothesis for the eight *Crotalaria* species, in order to test our last hypothesis.

## Materials and Methods

### Study System

The rattlebox moth *Utetheisa ornatrix* (Erebidae: Arctiinae) is primarily Neotropical and extends to warmer areas of the Nearctic region [20]. *U*. *ornatrix*, together with five species that occur only in the Galapagos Islands, are the extant *Utetheisa* species in the Neotropics [34,35]. The adults are generally found flying near patches of *Crotalaria* in pastures, the edge of woods and roadsides, where the larvae can be found feeding on both leaves or seeds inside the unripe pods of *Crotalaria* [21,26,28].

*Crotalaria* (Leguminosae: Papilionoideae: Crotalarieae) is a Pantropical genus of weeds, comprising approximately 702 species [22,36]. In the Neotropics, particularly in Brazil, 31 native and 11 non-native species have been recorded [37]. *Crotalaria* species are rich in PAs [34], which are found in higher concentrations in seeds than in leaves [26]. In addition to PAs, *Crotalaria* species have other defensive traits against herbivores including antifeeding deterrents such as lectins [38], non-protein amino acids [39] and protease inhibitors [40]. They also have extrafloral nectaries (EFNs) that attract predatory ants and wasps [26,41–43]. We used eight species of *Crotalaria*, including three natives, three non-natives, and two with uncertain origins (Table 1), that have been planted in an open area near the Animal Biology Department, Institute of Biology at the State University of Campinas, Campinas, São Paulo, Brazil (22°49'15.38"S, 047°04'8.87"W). In natural environments, we have observed *U*. *ornatrix* using *C*. *incana*, *C*. *micans*, *C*. *spectabilis*, *C*. *pallida* and *C*. *vitelline* as host plants. For the other three species, we have no information about their natural use by *U*. *ornatrix*.

**Table 1:** Species of *Crotalaria* used to feed larvae of *Utetheisa ornatrix* for *Nephila clavipes* bioassay and PA analysis.

Species	Section	Native range
*C*. *incana* L.	*Chrysocalycinae*	Pantropical (uncertain origin)^2^
*C*. *juncea* L.	*Calycinae*	India, Asia^2^
*C*. *micans* Link	*Chrysocalycinae*	Neotropics^1^ ^,^ ^2^
*C*. *ochroleuca* G. Don	*Hedriocarpae*	Tropical Africa^2^
*C*. *pallida* Aiton	*Hedriocarpae*	Pantropical (uncertain origin) ^2^
*C*. *paulina* Schrank	*Calycinae*	Neotropics^1^ ^,^ ^2^
*C*. *spectabilis* Roth	*Crotalaria*	Asia^2^
*C*. *vitellina* Ker Gawler	*Chrysocalycinae*	Neotropics^1^

^1^Flores [38]

^2^Polhill [22]

**Fig 1:**
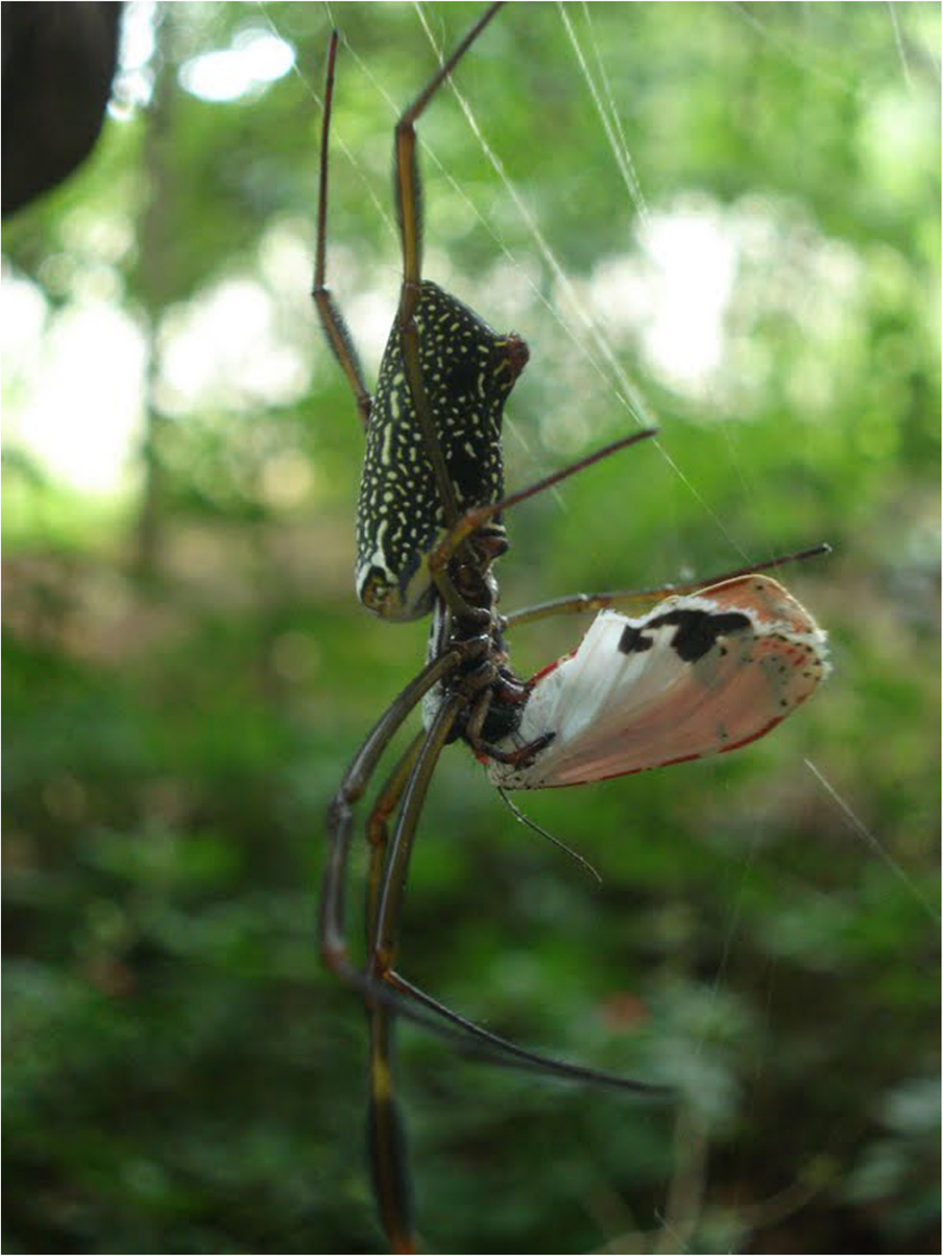
Female of *Nephila clavipes* handling an adult male of *Utetheisa ornatrix*. Note that the spider touches the moth with her pedipalps, probably evaluating the content of defensive pyrrolizidine alkaloids.

The neotropical orb-weaving spider *Nephila clavipes* (Nephilidae) is a predator that builds its web in forest clearings and corridors, which are flight paths for insects [44]. This spider preys on grasshoppers, bees, wasps, moths, and butterflies, but is able to discriminate PA-containing insects, releasing them unharmed [5] (Fig 1). In the edges of woods, this spider co-occurs with *U*. *ornatrix*.

The permit for research with wild animals was provided by IBAMA-ICMBio (Ministério do Meio Ambiente, Brazil).

### Rearing *Utetheisa ornatrix* Larvae

We obtained larvae of *U*. *ornatrix* from adults collected on the Fazenda Santa Mariana, Campinas, São Paulo, Brazil (22°47’02.91”S, 47°00’36.03”W), where the host plant *C*. *pallida* is abundant. We brought the adults to the laboratory, sexed them following Travassos [45], and allowed them to mate (20 males and 10 females per cage) in a paper cage (ca. 3.2 L) following Cogni [28]. We supplied food in a vial containing 5.0% aqueous honey. After 3–4 days, the adults started to oviposit on the paper-cage surfaces. The eggs were mixed to randomize the parental origin. After eclosion, we reared the first instars on leaves or unripe seeds of one of the eight species of *Crotalaria*. We also reared a group of first instars on a PA-free diet, following Cogni et al. [46]. For each treatment (diets of leaves or unripe seeds of each species of *Crotalaria*), we reared ca. 20 individuals in a plastic container (6 cm high, 20 cm diameter) until pupation. For some treatments, depending on the availability of seeds and leaves, we had 2 or 3 containers, giving 40–60 individuals per treatment. The larvae were fed *ad libitum*. We inspected the containers daily, removing feces and dead individuals, and replacing the leaves or seeds with new ones. We moved the pupae to another container with the same dimensions until adult emergence, since the larvae can cannibalize pupae [47]. We carried out the same procedure for pupae from the PA-free diet. We moved newly emerged adults to paper cages, separating them by treatment and sex, until the spider-predation bioassays. We kept both the paper cages with adults and the plastic containers with larvae and host plants, or pupae, at room temperature.

### Predation Bioassay with Living Moths

We carried out the bioassays with *Nephila clavipes* in a small patch of woods in Campinas (22°48'21.26"S, 47°4'43.12"W) from March to May in 2012 and 2013, when the spiders were abundant. We conduced all bioassays between 09:00 and 16:00 hs. We used only adult female spiders that responded immediately when any prey was tossed into their web. We did not use spiders that were in the course of feeding on a prey insect, but we did not control for satiation before each bioassay. We used around 80 spiders for the bioassays. Since the number of spiders was a limiting factor, sometimes we repeated the bioassays with the same spider, but each individual was used only once per week. We bioassayed 481 adult moths, testing around 20 individuals for each sex, host plant and plant part. We made a small cut in the moth's hind wings before placing it on the web, to prevent the moth from flying away if released by the spider. We placed the moth on the web, and recorded if it was eaten or released. We considered the spider response to be predation when it bit, wrapped and killed the moth. When the spider cut the web around the moth after touching it and freed it unharmed, we recorded the response as a release. We did not record the rejection behavior described by Vasconcellos-Neto and Lewinsohn [48] (the prey was initially sucked and then freed), since the prey was removed from the web before this stage. If the spider released the moth, we offered a palatable freeze-killed mealworm *Tenebrio molitor* (Coleoptera: Tenebrionidae) as a control. We recorded the trial as a rejection only if the spider fed on the mealworm. If the spider killed the experimental moth, we did not use the control. When predation was recorded, we immediately removed the moth from the web and placed it in a 1.5-mL Eppendorf tube filled with MeOH for further PA analysis. All moths were recovered intact; even the parts bitten by the spider had no visible signs of damage. We also preserved the released moths in MeOH as above. We analyzed 310 individuals; 171 were not used for PA analysis, since they were lost after the spider bioassay.

### Pyrrolizidine Alkaloids: Extraction, Quantification, Characterization and Isolation

To quantify the PAs, we extracted the freeze-dried samples of unripe seeds, leaves of host plants or adult moths three times with EtOH (10x volume:weight). We centrifuged the EtOH extract at 10,416 rcf for 10 min, and recovered the EtOH layer. We completed the EtOH layer to 20 mL and took an aliquot (0.1 or 1.0 mL) to carry out the colorimetric analysis according to Trigo et al. [49,50]. We used monocrotaline for the reference curve. The total PA concentration was given in μg of PAs/mg of dry weight.

For PA characterization by gas chromatography-mass spectrometry (GC-MS), we extracted the plant or insect samples using the acid-base procedures described by Trigo et al. [49,50]. The GC-MS analysis was carried out in electron impact mode according to Flores et al. [29]. The retention indices and mass fragmentation patterns were compared with published descriptions (see Table 2, and references [29,51,52]).

**Table 2:** Mass fragmentation pattern of pyrrolizidine alkaloids found in *Crotalaria* species and in adults of *Utetheisa ornatrix* fed on these host plants. The analyses were carried out by gas chromatography-mass spectrometry (GC-MS) in electron impact mode and liquid chromatography-mass spectrometry (LC-MS) in electrospray ionization mode.

Pyrrolizidine alkaloids	RI^a^	Rt^b^	Diagnostic ions for GC-MS ^c^, m/z (%)	Diagnostic ions for LC-MS^d^, m/z (%)	Reference for GC-MS^e^
Retronecine	1487	6.085	[M^+^] 155 (23), 111 (60), 94 (17), 80 (100)	[2M+H]^+^ 343 (9), [M+H]^+^ 172 (100)	[29]^1^
9-(2^’^-Hydroxy)-ethanoylretronecine-like	1795	nd	[M^+^] 227 (2), 183 (6), 138 (62), 120 (6), 93 (100), 80 (40)	nd	[29]^1^
9-(2-Hydroxy-3-methylpentanoyl)-trachelanthamidine	1831	nd	[M]^+^ 255 (2), 142 (6), 125 (10), 124 (100), 96 (4), 83 (20), 82 (12)	nd	[52]^1^
Creatonotine B-like	1840	nd	[M]^+^ 255 (3), 211 (7), 138 (99), 124 (13), 120 (11), 94 (40), 93 (100), 80 (25)	nd	[51]^2^
7-Senecioyl retronecine-like	1861	nd	[M]^+^ 237 (5), 137 (29), 136 (18), 94 (25), 93 (10), 80 (100)	nd	[29]^1^
9-Senecioyl retronecine-like	1890	nd	[M]^+^ 237 (3), 193 (9), 154 (15), 138 (27), 137 (28), 136 (20), 94 (24), 93 (100), 80 (20)	nd	[29]^1^
Iso-creatonotine B	2024	22.075	[M]^+^ 269 (3), 251 (10), 138 (40), 124 (17), 120 (26), 111 (61), 106 (51), 94 (26), 80 (100)	[M+Na]^+^ 308 (11), [M+H]^+^ 286 (100)	[51]^1^
7-Octanoyl retronecine-like	2031	nd	[M]^+^ 281 (50), 220 (19), 154 (8), 136 (22), 124 (22), 111 (69), 106 (47), 94 (24), 80 (100)	nd	[29]^1^
Creatonotine B	2048	22.969	[M]^+^ 269 (<1), 251 (1), 225 (5), 138 (100), 93 (88), 80 (18)	[M+Na^+^] 308 (6), [M+H]^+^ 286 (100)	[51]^1^
9-Octanoyl retronecine-like	2052	nd	[M]^+^ 281 (5), 236 (6), 138 (62), 120 (26), 106 (13), 94 (47), 93 (100), 80 (44)	nd	[29]^2^
1,2-Dihydrocreatonotine B	2064	nd	[M]^+^ 271 (4), 210 (12), 171 (20), 140 (25), 139 (11), 114 (10), 96 (34), 95 (70), 82 (100)	nd	[52]^1^
9-(5’-Hydroxy)-heptanoylretronecine-like	2082	24.162	[M]^+^ 283 (1), 224 (4), 155 (24), 138 (65), 93 (100), 80 (19)	[M+Na]^+^ 322 (14), [M+H]^+^ 300 (100)	[29]^1^
Crispatine-like	2175	22.598	[M]^+^ 309 (2), 222 (7), 136 (88), 120 (83), 119 (100), 93 (62), 80 (29)	[M+Na]^+^ 348 (11), [M+H]^+^ 326 (100)	[29]^3^
Unknown monocrotaline-type	2243	24.936	[M]^+^ 323 (3), 236 (4), 208 (10), 136 (97), 120 (87), 119 (100), 93 (54), 80 (27)	[M+Na]^+^ 362 (9), [M+H]^+^ 340 (100)	[29]^3^
Incanine-like	2315	27.280	[M]^+^ 337 (6), 264 (11), 250 (5), 222 (8), 136 (100), 120 (76), 119 (79), 93 (49), 80 (27)	[M+Na^+^] 376 (15), [M+H^+^] 354 (100)	[29]^1^
Monocrotaline	2336	18.610	[M^+^] 325 (1), 254 (3), 236 (46), 136 (52), 120 (100), 93 (38), 80 (19)	[M+Na]^+^ 364 (6), [M+H]^+^ 342 (100)	[29]^1^
Senecionine / Integerrimine	2339/2410	26.789/ 25.111	[M]^+^ 335 (6), 291 (12), 248 (12), 220 (21), 136 (97), 120 (100), 93 (83), 80 (34)	[M+Na)^+^] 374 (13), [M+H]^+^ 352 (100)	[29]^1^
Trichodesmine-like	2341/2348	25.326	[M]^+^ 353 (6), 264 (100), 136 (31), 120 (35), 93 (44), 80 (14)	[M+Na]^+^ 392 (17), [M+H]^+^ 370 (100)	[29]^1^
Unknown monocrotaline-type	2346	26.050	[M]^+^ 337 (2), 222 (5), 136 (58), 120 (89), 119 (100), 93 (40), 80 (18)	[M+Na]^+^ 376 (20), [M+H]^+^ 354 (100)	[29]^3^
Unknown monocrotaline-type	2346	nd	[M]^+^ 353 (2), 264 (45), 136 (75), 120 (100), 93 (70), 80 (25)	nd	[29]^2^
Senecionine-like	2376	nd	[M]^+^ 337 (6), 222 (18), 136 (75), 120 (100), 93 (67), 80 (32), 55 (47)	nd	[29]^2^
Methylmonocrotaline-like	2384	nd	[M]^+^ 339 (1), 250 (58), 136 (53), 120 (100), 93 (39), 80 (20)	nd	[29]^1^
Senecionine-like	2386	nd	[M]^+^ 321 (13), 247 (7), 136 (98), 120 (100), 93 (76), 80 (37)	nd	[29]^1^
Trichodesmine-like	2418	23.415	[M]^+^ 353 (2), 264 (60), 136 (47), 120 (100), 93 (35), 80 (18)	[M+Na]^+^ 392 (13), 370 [M+H]^+^ 370 (100)	[29]^1^
Incanine-like	2430	nd	[M]^+^ 337 (5), 264 (27), 222 (20), 136 (62), 120 (100), 93 (65), 80 (27)	nd	[29]^1^
Trichodesmine-like	2437	nd	[M]^+^ 353 (2), 264 (83), 222 (8), 136 (58), 120 (100), 93 (34), 80 (18)	nd	[29]^1^
Senecionine-like	2540	nd	[M]^+^ 337 (1), 155 (12), 138 (71), 136 (28), 93 (100), 80 (19), 55 (27)	nd	[29]^2^
Senecionine-like	2551	nd	[M]^+^ 351 (31), 220 (10), 136 (43), 120 (100), 119 (90), 93 (75), 80 (33)	nd	[29]^2^
Platyphorine C-like	2556	25.260	[M]^+^ 383 (2), 281 (13), 267 (11), 252 (100), 138 (24), 136 (27), 120 (51), 93 (51), 80 (18)	[M+Na]^+^ 422 (19), [M+H]^+^ 400 (100)	[52]^1^
Unknown *seco*-PA	2615	nd	[M]^+^ 365 (20), 321 (15), 276 (22), 238 (100), 168 (70), 122 (38), 110 (32), 94 (31), 83 (46)	nd	[29]^1^
Retrorsine/Usaramine	2621/2647	22.989/ 22.731	[M]^+^ 351 (7), 246 (5), 136 (100), 120 (99), 119 (84), 93 (80), 80 (35)	[M+Na]^+^ 390 (7), [M+H]^+^ 368 (100)	[29]^1^
Senecionine-like	2675	nd	[M]^+^ 351 (3), 224 (8), 143 (100), 136 (65), 120 (86), 119 (67), 93 (51), 80 (20)	nd	[29]^2^
Senecionine-like	2684	nd	[M]^+^ 351 (2), 143 (24), 136 (51), 120 (100), 119 (91), 93 (79), 80 (23)	nd	[29]^2^
Unknown *seco*-PA	2728	nd	[M]^+^ 381 (25), 338 (55), 320 (63), 250 (58), 238 (77), 168 (100), 150 (32), 122 (52), 110 (44)	nd	[29]^1^
Unknown *seco*-PA	2815	nd	[M]^+^ 379 (43), 334 (26), 238 (22), 168 (74), 151 (29), 139 (57), 122 (100), 110 (62), 94 (57)	nd	[29]^1^
Unknown *seco*-PA	2866	nd	[M]^+^ 421 (31), 376 (22), 168 (77), 150 (79), 139 (67), 122 (100), 110 (64), 94 (45), 43 (96)	nd	[29]^1^
Unknown *seco*-PA	2907	nd	[M]^+^ 437 (34), 250 (79), 226 (41), 197 (78), 183 (78), 168 (44), 122 (100), 110 (52), 94 (52)	nd	[29]^1^

^a.^ Retention index in GC-MS analyses.

^b.^ Retention time (min) in LC-MS analyses. Some compounds detected by GC-MS were not detected (nd) by LC-MS.

^c.^ in free base form.

^d.^ in *N*-oxide form.

^e. Characterization of PAs by mass spectra using: 1^Mass spectra matching with literature, ^2.^Interpretation of the mass spectrum from literature, ^3^Erroneously described in 29 as unknown senecionine-type.

For PA characterization by liquid chromatography-mass spectrometry the samples were extracted in EtOH as above. The EtOH layer was evaporated in a rotaevaporator at 40°C, recovered with 1.5 mL 2% aqueous acetic acid and cleaned 3 times with the same volume of hexane. The acid solution was added to a clean 2-mL vial, capped, and stored at -20°C until LC-MS analysis. We used an Agilent 1260 Infinity Quaternary LC, equipped with an Eclipse Plus C-18 column, 4.6 x 250 mm, 5 μm, and a guard column with the same phase, coupled with an Agilent 6130 single quadrupole in electrospray ionization mode. The column was kept at 40°C, and the injection volume was 5 to 50 μl. The PA was separated using a linear gradient containing aqueous 20 mM ammonium acetate and MeOH at flow rate 0.5 mL/min. The gradient started at 95% ammonium acetate:5% MeOH (3 min), MeOH was raised to 100% in 25 min, and kept at 100% for 3 min. The mass spectrometer was run in positive mode, scanning the product ions from 100–500 amu (see Table 2).

**Fig 2:**
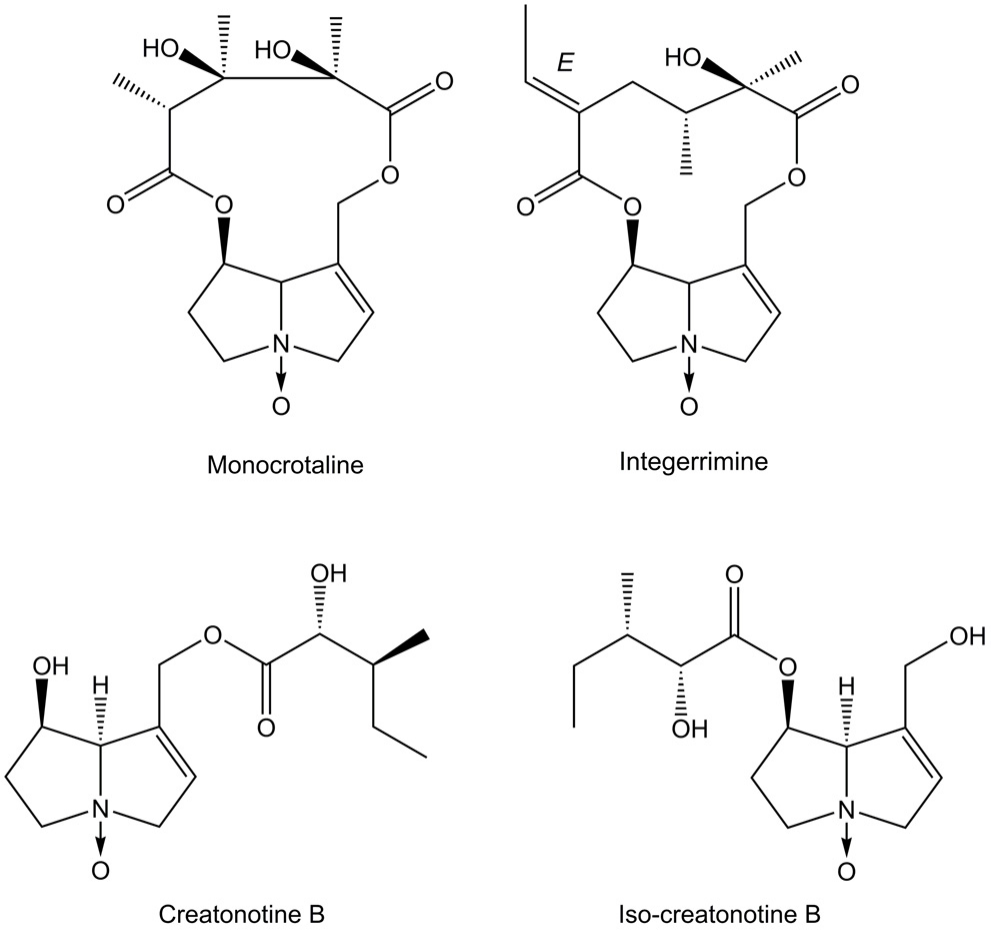
Some pyrrolizidine alkaloids found in this study. The alkaloids are drawn in their *N*-oxide form. For more structures, see, e.g., Flores et al. [29].

For the predation bioassays with pure PAs in *N*-oxide form, we isolated monocrotaline from *C*. *spectabilis*, integerrimine from *Senecio brasiliensis* (Asteraceae: Senecioneae) and the putative mixture insect PAs creatonotine B: iso-creatonotine B (Fig 2) from adults of *U*. *ornatrix* that fed as larvae on leaves and unripe seeds of *C*. *vitellina*. We extracted PAs using the acid-base procedure as described above, and isolated them using an adsorption column chromatograph (40 cm length, 2.5 cm diameter), using Silica Gel 70–325 mesh as the stationary phase, and a gradient from CHCl_3_ to CHCl_3_:MeOH:Et_3_N 85:14:1 as the mobile phase. We followed the PAs in 10-mL fractions by silica-gel thin-layer chromatography with CHCl_3_:MeOH:NH_4_OH 85:14:1 as eluent and Dragendorff's reagent for detection. We *N*-oxidized the PAs and purified them using the procedure described by Craig and Puroshothaman [53].

### Predation Bioassay with Pure Pyrrolizidine Alkaloids

We found that adults of *U*. *ornatrix* generally contained PA *N*-oxides of monocrotaline and integerrimine types, and the specific mixture of insect PAs creatonotine B: iso-creatonotine B (see Results). We carried out predation bioassays with *N*. *clavipes* to determine if PA *N*-oxides with different structures had similar activity against the spider. The bioassays were carried out in the same area where we carried out the predation bioassay with living moths.

We treated palatable freeze-killed larvae of the mealworm *T*. *molitor* (hereafter baits) with 3.0 μg/mg dry weight of each alkaloid, which were obtained as described above. This concentration was determined by calculating the logistic regression of the *N*. *clavipes* response in relation to PA concentration in adults of *U*. *ornatrix* fed as larvae on one of the eight different host plants (see Statistical analyses), where at 3.0 μg/mg the probability of release by the spider was 87%. To evaluate whether the PA concentration also played any role in the chemical protection against the spider, we also bioassayed these PA *N*-oxides in a one-tenth concentration. Additionally, we conducted bioassays with 5.0 μg/mg *N*-oxide for the insect PA. All bioassays were conducted using 10 baits for each PA concentration. The PAs were diluted in MeOH and applied topically with a 10-μL syringe on the surface of the bait, which was killed by freezing. We used a MeOH-treated bait as a control to determine whether to accept the trial, as described above for the *N*. *clavipes* predation bioassay with living moths.

### Host Plant Phylogenetic Hypothesis and Character Mapping

We mapped the characters of PAs involved in the chemical defenses of *U*. *ornatrix* in an independent phylogenetic hypothesis for the eight *Crotalaria* species. This procedure may reveal the evolutionary trends of mapped characters [54]. We did this only for those that were fed on leaves, as all individuals fed on seeds were well protected against the spider, regardless of the host plant. As PA characters we used PA concentration (high > 3.0 μg/mg and low < 1.0 μg/mg) and PA type (monocrotaline, senecionine, senkerkine or insect PA) in all eight *Crotalaria* species and *U*. *ornatrix*, and the response of the predator *N*. *clavipes* in relation to *U*. *ornatrix* (percentage of release).

We constructed a phylogenetic hypothesis based on ITS sequences retrieved from GenBank for seven *Crotalaria* species and for the out-group *Bolusia amboensis* [36]. Total genomic DNA of *C*. *vitellina* (unavailable in GenBank) was extracted from fresh leaves, collected in the garden of UNICAMP, using the two-fold hexadecyltrimethylammonium bromide (CTAB) method [55]. The nuclear ribosomal ITS region (ca. 700 bp) was amplified as described by le Roux et al. [36], using the primers ITS 17SE and ITS 26SE [56,57]. PCR products were purified with a GFX PCR DNA and Gel Band Purification Kit (GE Healthcare) and sequenced at the Center of Molecular Biology and Genetic Engineering, UNICAMP, using an automated capillary sequencer (ABI PRISM 3700 DNA Analyzer, Applied Biosystems). Complementary sequences were assembled and edited with the Muscle algorithm [58] in MEGA 6 [59]. The *C*. *vitellina* ITS sequence was submitted to GenBank (KR013000). This sequence was aligned visually with the sequences gathered in GenBank, using MEGA 6, and the most appropriate nucleotide substitution model for the set of sequences was determined with the same software. Phylogenetic analyses were carried out using Bayesian Inference (maximum posterior probability, MPP) in Beast v1.8.1 [60] for a total of 10 million generations, with tree sampling every 1 million generations. A Yule speciation process was assumed, as recommended for species-level phylogenies [61]. The HKY+I substitution model was used for the substitution rate, and the default prior distribution was used for all other parameters. Tracer v.1.6 [62] was used to assess convergence using a minimum ESS value of 200. After the analyses were completed, 10% of the trees were removed as “burn-in”. The tree was visualized in FigTree v.1.4.2 (http://tree.bio.ed.ac.uk/software/figtree/).

### Statistical Analysis

We analyzed the frequency of individuals predated or released, using a generalized linear model (GLM) with binomial distribution and logit function link, using the package “bbmle” in R 3.1.0 for Windows [63,64]. Different models were generated to assess the effects of the explanatory variables (host plant, plant part, and sex of moth) as well as interactions between and among these variables in the response variable frequency of individuals predated or released. The model host plant, host plant part, and interaction between these factors provided the lowest AIC (Akaike’s Information Criterion) value (= 0.0). Therefore, we used an 8 x 2 design, where the factors were host plants (eight levels) and the part of the host plants where the larvae fed (two levels).

We compared the response variable concentration of PAs (μg/mg) in moths between parts of plant (two levels) and host-plant treatments (eight levels) by a two-way ANOVA, using the Tukey post-hoc test [65]. We discharged the explanatory variable sex, using AIC as above. We ln-transformed the PA concentration (in μg/g to avoid values < 1 in the ln transformation) to meet ANOVA assumptions [65]. A similar analysis was used to compare the PA concentration in the host plants. We calculated the nonlinear relationship between the PA concentration in *U*. *ornatrix* and the PA concentration in the leaves or seeds of the host plants, using the CurveExpert Professional 2.2.0.

We assessed the relationship between the *N*. *clavipes* response (predation or release) for individual adults of *U*. *ornatrix* feeding on each of eight different host plants and their PA concentrations, by a simple logistic regression [65]. We also tested this relationship after pooling the data for adults feeding on all host plants.

We asked if there is a relationship between the PA concentration in adults whose larvae fed on leaves or seeds and *N*. *clavipes* release, and if this relationship depended on the species of *Crotalaria*. We calculated this nonlinear relationship, as described above, using the mean PA concentration in adults whose larvae fed on leaves or seeds of each host plant and the percentage of adults released by the spider for each host plant.

As adults reared on different *Crotalaria* species showed a different PA profile with three PA classes (monocrotaline, senecionine and insect PA), we compared the response-variable percentage of adults released by *N*. *clavipes* in relation to the explanatory variables different PA structures (two levels) and concentration (two levels), topically applied to a palatable bait. We used a GLM binomial analysis as described above.

## Results

### Predation Bioassay with Living Moths

**Fig 3:**
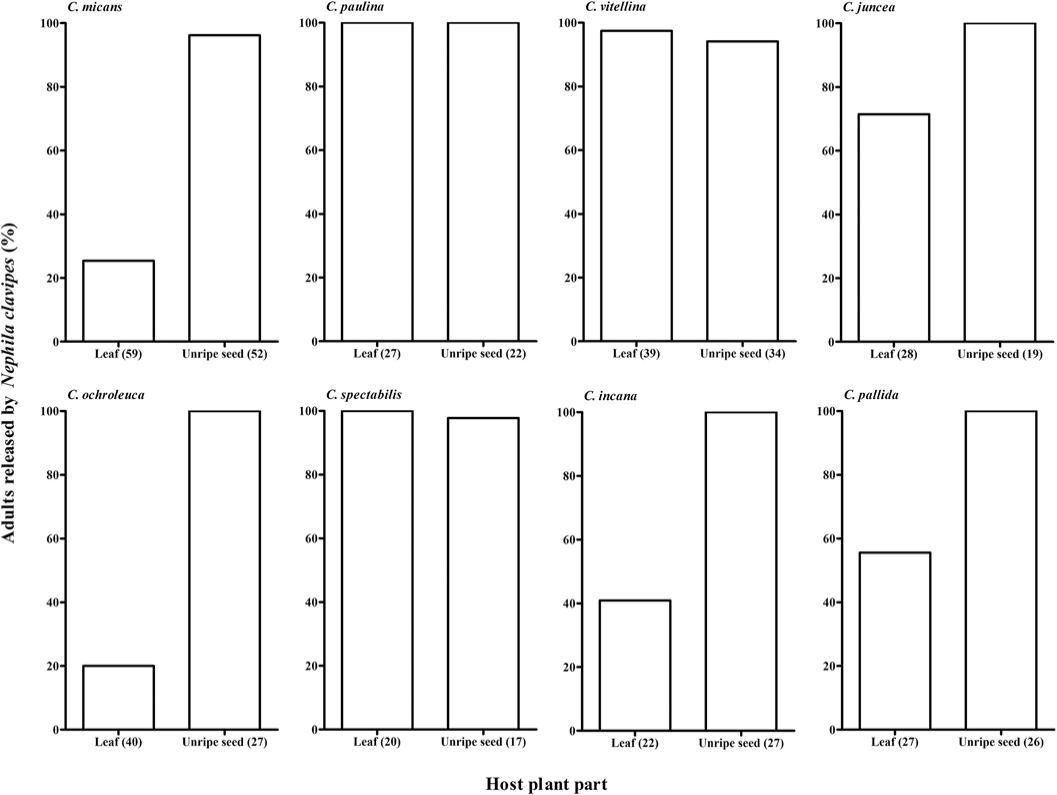
Percentage of adults of *Utetheisa ornatrix* released by *Nephila clavipes* with respect to eight host-plant species and plant parts (leaf or unripe seed) fed to the larvae. The natives *Crotalaria micans*, *C*. *paulina*, and *C*. *vitellina*, non-natives *C*. *juncea*, *C*. *ochroleuca*, and *C*. *spectabilis*, and species of uncertain origin *C*. *incana* and *C*. *pallida*. Numbers in parentheses in "plant part" represent the number of adults bioassayed for each diet.

The frequency of predation or release was affected by the host plant (GLM binomial, χ^2^ = 104.714, df = 7, P <0.001), the part of the plant consumed by the larvae (χ^2^ = 147.355, df = 1, P <0.001) and by the interaction between these factors (χ^2^ = 19.274, df = 7, P = 0.007). Across all eight hosts, the spiders released 57.2% of the moths whose larvae fed on leaves, whereas 97.8% of the moths whose larvae fed on unripe seeds were released. The release percentage was lower for leaf feeders compared to seed feeders, for adults when larvae fed on *C*. *incana*, *C*. *micans*, *C*. *juncea*, *C*. *ochroleuca* and *C*. *pallida* (Fig 3). Adults whose larvae fed on either leaves or seeds of *C*. *paulina*, *C*. *vitellina* and *C*. *spectabilis* were equally well protected against *N*. *clavipes* attacks; almost all individuals were released (Fig 3).

### Pyrrolizidine Alkaloids: Sequester from Larval Host Plants and Transformation

#### Pyrrolizidine alkaloid concentration in moth and host plants

**Fig 4:**
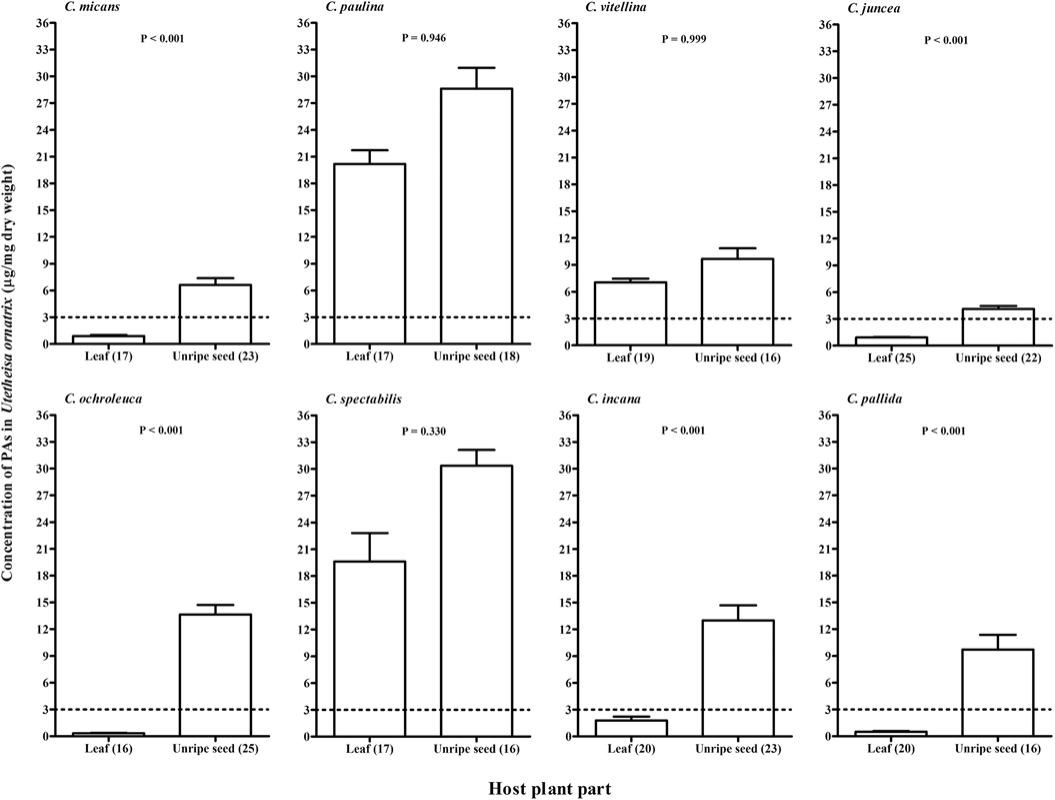
Concentration of pyrrolizidine alkaloids (mean ± se) in adults of *Utetheisa ornatrix* with respect to eight host-plant species and plant parts (leaves or unripe seeds) fed to the larvae. Numbers in parentheses in "plant part" represent the number of adults analyzed for each diet. The dotted line is the concentration at which baits treated with monocrotaline and integerrimine in the *N-*oxide form (3.0 μg/mg) were 100% released by *Nephila clavipes*. The probability that the PA concentration in adults fed as larvae on leaves or seeds, for each host plant, is significantly different is given above the bars (*post hoc* Tukey test). For other statistics, see Results.

The concentration of PAs differed among adults whose larvae fed on different host plants (two-way Anova, F_7,294_ = 118.50, P < 0.001). Adults feeding as larvae on *C*. *paulina* and *C*. *spectabilis* had higher concentrations of PAs compared with adults reared on other species (Tukey test P < 0.001, Fig 4). The concentrations of PAs in adults also differed between plant parts fed to the larvae (F_1,294_ = 585.73, P < 0.001). The concentration of PAs was significantly higher in larvae feeding on seeds than in those feeding on leaves, but for larvae fed on *C*. *paulina*, *C*. *spectabilis* and *C*. *vitellina* we found no significant differences (Fig 4). We found an interaction between host plants and plant parts (F_7,294_ = 39.51, P < 0.001). Adults whose larvae fed on leaves of *C*. *paulina*, *C*. *spectabilis* and *C*. *vitellina* showed significantly higher PA concentrations than those reared on leaves of other *Crotalaria* species (Tukey test, P<0.001, Fig 4); however, larvae fed on *C*. *vitellina* showed a significantly lower concentration than both *C*. *paulina* and *C*. *spectabilis* feeders (P < 0.001, Fig 4).

**Fig 5:**
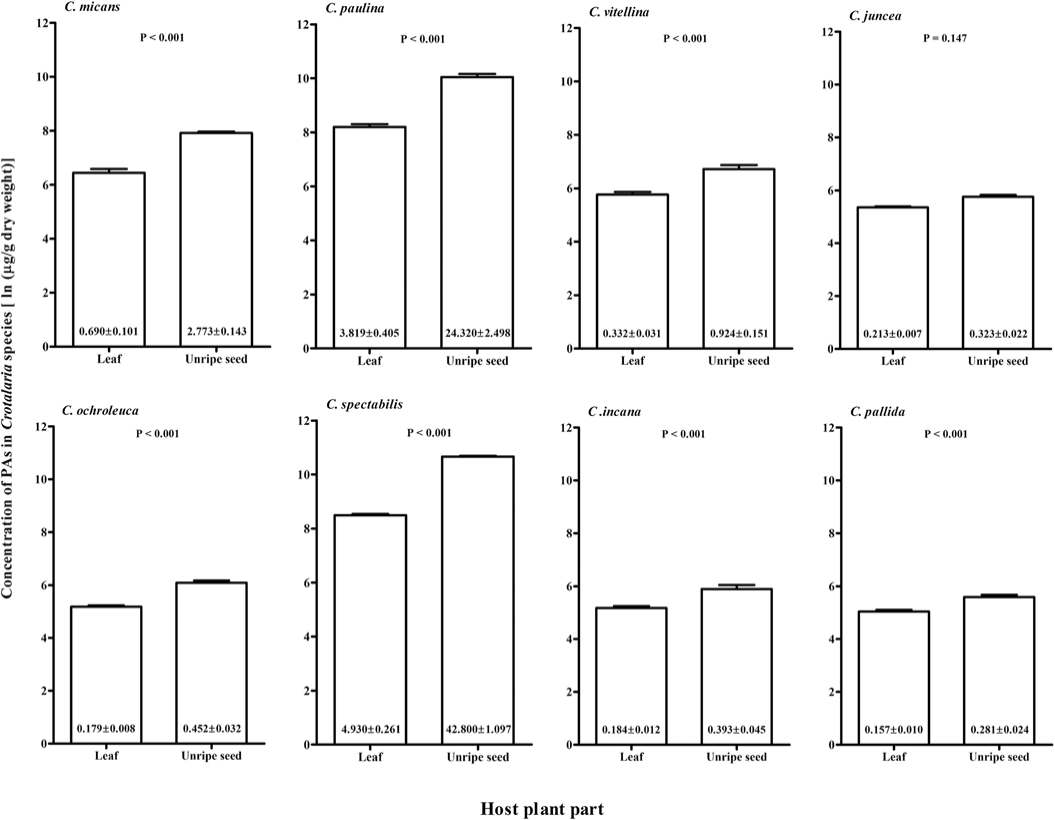
Concentration of pyrrolizidine alkaloids (mean ± se) in leaves and unripe seeds of *Crotalaria* species, which were used to rear *Utetheisa ornatrix* larvae. The concentration is given in ln transformed data (μg/g dry weight). The numbers inside the bars represent the mean ± SE of untransformed data in μg/mg dry weight. The probability that the PA concentration in leaves and seeds, for each host plant, is significantly different is given above the bars (*post hoc* Tukey test).

The PA concentrations in host plants differed significantly among the *Crotalaria* species (two-way Anova, F_7,149_ = 678.2, P < 0.001), and were highest in *C*. *paulina* and *C*. *spectabilis* (Fig 5). The concentration of PAs was significantly higher in seeds than in leaves (F_1,149_ = 596.2, P < 0.001, Fig 5). A significant interaction between host species and host plant part occurred (F_7,149_ = 23.8, P < 0.001), due to the lack of a significant difference between plant parts for *C*. *juncea* (Tukey test, P = 0.147).

**Fig 6:**
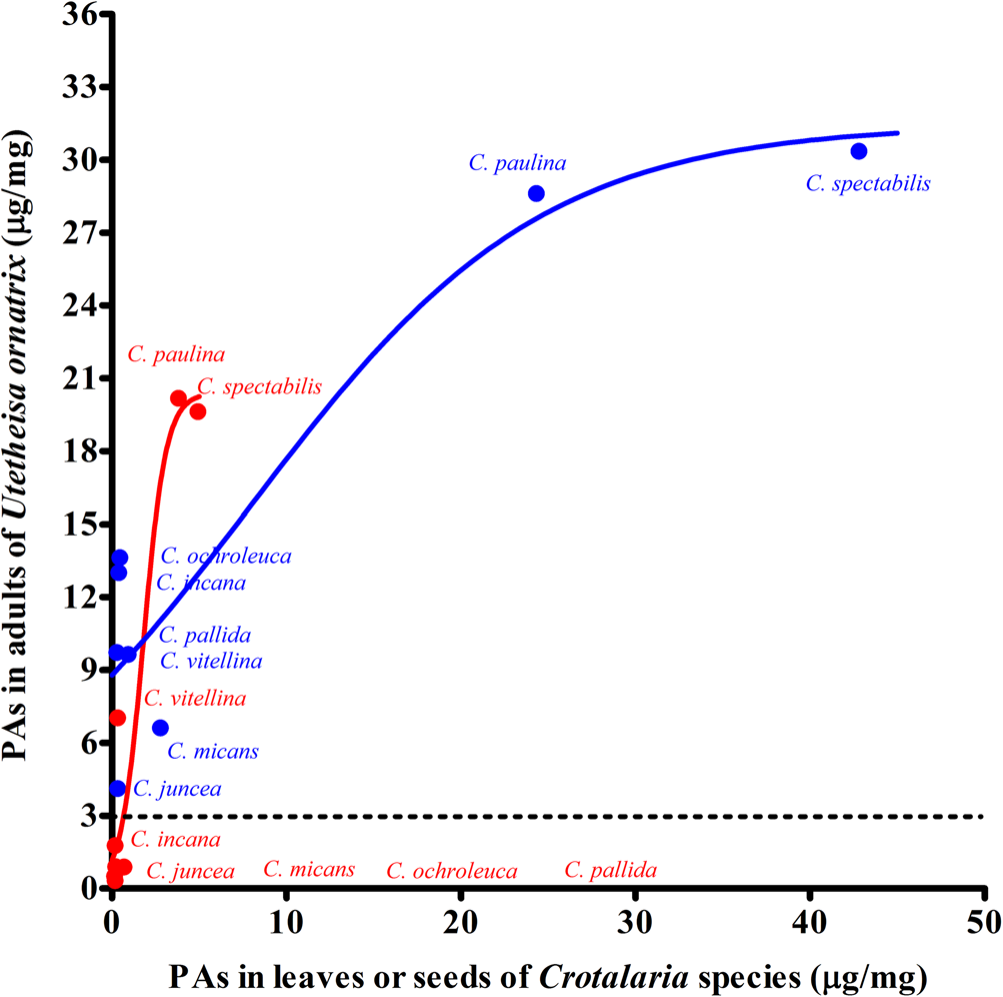
Non-linear relationship between the mean of pyrrolizidine alkaloid concentration (μg/mg dry weight) in *Crotalaria* species and in adults of *Utetheisa ornatrix*. Red symbols are leaves and blue are seeds. The dotted line is the concentration at which baits treated with monocrotaline and integerrimine in the *N-*oxide form (3.0 μg/mg) were 100% released by *Nephila clavipes*.

There was a positive relationship between the PA concentration in *Crotalaria* species and *U*. *ornatrix*, regardless of the part of the plant that the larvae fed on [nonlinear regression for larvae fed on leaves y = 20.39/(1+16.48e^-1.55x^), r^2^ = 0.93, and for larvae fed on unripe seeds y = 31.47/(1+2.58e^-0.11x^), r^2^ = 0.88, Fig 6]. However, we noted a maximum PA sequestration in *U*. *ornatrix* (around 30 μg/mg), independently of an increase in the concentration of PAs in their host-plant seeds.

#### Pyrrolizidine alkaloid sequestration and transformation

The transformation of sequestered PAs into insect PAs is host-plant dependent. Adults whose larvae fed on host plants with monocrotaline-type (*C*. *juncea*, *C*. *paulina* or *C*. *spectabilis*) or integerrimine-type PAs (*C*. *incana* or *C*. *ochroleuca*) sequestered a large amount of unchanged alkaloids; small amounts of biosynthesized insect PAs were also found (Table 3). Adults whose larvae fed on *C*. *vitellina*, a host plant whose alkaloids are mainly seco-PAs (alkaloids with an otonecine base, such as senkirkine) and the necine base retronecine, biosynthesize large amounts of insect PA iso-creatonotine and creatonotine B; the seco-PAs, which are not *N*-oxidized, were not sequestered, but eliminated in the feces (data not shown) (Table 3). Adults whose larvae fed on *C*. *micans* or *C*. *pallida* showed high amounts of insect PAs together with unchanged PAs originating from the host plant; *C*. *micans* contained retronecine and integerrimine as the main PAs, and *C*. *pallida* contained usaramine (Table 3). All PAs in moths and seeds were present in the *N*-oxide form, except for the seco-PAs in *C*. *vitellina* and 34.7% of the monocrotaline in *C*. *spectabilis*, which was present in free base form.

**Table 3:** Relative abundance (%) of pyrrolizidine alkaloids in adults of *Utetheisa ornatrix* (male: M, female: F) and the species of *Crotalaria* used as larval host plants (HP). *C*. *micans*: mic; *C*. *paulina*: pau; *C*. *vitellina*: vit; *C*. *juncea*: jun; *C*. *ochroleuca*: och; *C*. *spectabilis*: spe; *C*. *incana*: inc; *C*. *pallida*: pal. Five individuals were used for GC-MS analysis. Only seeds were analysed; the alkaloid profile of leaves was similar. The data are shown as mean ± standard error.

Pyrrolizidine alkaloids	RI	mic M	mic F	mic HP	pau M	pau F	pau HP	vit M	vit F	vit HP	jun M	jun F	jun HP	och M	och F	och HP	spe M	spe F	spe HP	inc M	inc F	inc HP	pal M	pal F	pal HP
Retronecine	1487	19±2	22±3	21±6	-	2±1	-	26±2	32±4	3±1	-	2±1	-	6± 1	8±1	2±1	-	-	-	3±1	6±1		13±1	16±1	-
9-(2’-Hydroxy)-ethanoyl retronecine-like	1795	-	-	-	-	2±1	2±1	-	-	-	9±2	4±1	3±1	-	-	-	-	-	-	-	-	-	-	-	-
9-(2’-Hydroxy-3’-methylpentanoyl)-trachelanthamidine	1831	-	-	-	-	-	-	-	-	-	-	-	-	-	-	-	-	-	-	2±1	-	-	2±1	-	-
Creatonotine B-like	1840	-	-	-	-	-	-	-	-	-	-	-	-	-	-	-	-	-	-	-		-	-	1±1	-
7-Senecioyl retronecine-like	1861	-	-	3±1	-	-	-	-	-	-	-	-	-	-	-	2±1	-	-	-	-	-	2±1	-	-	-
9-Senecioyl retronecine-like	1890	-	-	10±2	-	-	-	-	-	-	-	-	-	-	-	2±1	-	-	-	-	-	3±1	-	-	-
Iso-creatonotine B	2024	9±1	13±3	-	-	-	-	9±1	10±1	-	-	-	-	2±1	2±1	-	-	1±1	-	-	1±1	-	5±1	5±1	-
7-Octanoyl retronecine-like	2031	-	-	-	2±1	1±1	-	-	-	-	6±1	11±4	13±1	-	-	-	-	-	-	-	-	-	-	-	-
Creatonotine B	2048	42±4	38±5	-	5±1	3±1	-	61±2	56±3	-	3±1	3±1	-	8±2	4±2	-	-	4±1	-	6±2	5±1	-	20±1	22±1	-
9-Octanoyl retronecine-like	2052	-	-	-	-	-	-	-	-	-	8±1	10±1	7±1	-	-	-	-	-	-	-	-	-	-	-	-
1,2 Dihydrocreatonotine B	2064	-	-	-	-	-	-	-	-	-	-	-	-	-	-	-	-	-	-	1±1	-	-	-	-	-
9-(5’-Hydroxy)-heptanoyl retronecine-like	2082	-	-	-	-	-	-	-	-	-	21±3	24±3	14±1	-	-	-	-	-	-	-	-	-	-	-	-
Desoxymonocrotaline	2175	-	-	-	17±1	21±1	42±2	-	-	-	-	-	-	-	-	-	-	-	-	-	-	-	-	-	-
Unknown monocrotaline-type	2243	-	-	-	38±2	52±1	29±2	-	-	-	-	-	-	-	-	-	-	-	-	-	-	-	-	-	-
Incanine-like	2315	-	-	-	-	-	-	-	-	-	6±1	2±1	12±1	-	-	-	-	-	-	-	-	-	-	-	-
Monocrotaline	2336	-	-	-	18±2	9±1	15±1	-	-	-	-	-	-	-	-	-	100	95±1	100	-	-	-	-	-	-
Senecionine	2339	3±1	4±2	-	-	-	-	-	-	-	-	-	-	-	-	9±1	-	-	-	-	-	-	1±1	2±1	1±1
Trichodesmine-like	2341	-	-	-	-	-	-	-	-	-	11±1	14±2	16±1	-	-	-	-	-	-	-	-	-	-	-	-
Methylmonocrotaline	2346	-	-	-	2±1	1±1	5±1	-	-	-	-	-	-	-	-	-	-	-	-	-	-	-	-	-	-
Trichodesmine-like	2348	-	-	-	-	-	-	-	-	-	20±1	16±1	17±1	-	-	-	-	-	-	-	-	-	-	-	-
Senecionine-like	2376	-	-	-	-	-	-	-	-	-	-	-	-	-	-	-	-	-	-	-	-	8±2	-	-	-
14-Methylmonocrotaline-like	2384	-	-	-	18±2	9±2	7±1	-	-	-	-	-	-	-	-	-	-	-	-	-	-	-	-	-	-
Senecionine-like	2386	-	-	-	-	-	-	-	-	-	-	-	-	-	-	-	-	-	-	-	-	-	1±1	1±1	-
Integerrimine	2410	22±6	17±4	66±4	-	-	-	-	-	-	-	-	-	65±4	70±2	50±1	-	-	-	53±3	87±1	76±4	1±1	-	7±1
Trichodesmine-like	2418	-	-	-	-	-	-	-	-	-	5±1	5±1	6±1	-	-	-	-	-	-	-	-	-	-	-	-
Incanine-like	2430	-	-	-	-	-	-	-	-	-	2±1	1±1	5±1	-	-	-	-	-	-	-	-	-	-	-	-
Trichodesmine-like	2437	-	-	-	-	-	-	-	-	-	9±1	8±2	7±2	-	-	-	-	-	-	-	-	-	-	-	-
Senecionine-like	2540	-	-	-	-	-	-	-	-	-	-	-	-	-	-	-	-	-	-	-	-	6±1	-	-	-
Senecionine-like	2551	-	-	-	-	-	-	-	-	-	-	-	-	-	-	-	-	-	-	-	-	-	2±1	2±1	-
Platyphorine C-like	2556	5±1	6±1	-	-	-	-	4±1	2±1	-	-	-	-	-	-	-	-	-	-	-	-	-	-	-	-
Unknown *seco*-PA	2615	-	-	-	-	-	-	-	-	8±1	-	-	-	-	-	-	-	-	-	-	-	-	-	-	
Retrorsine	2621	-	-	-	-	-	-	-	-	-	-	-	-	15±1	13±2	30±1	-	-	-	-	-	-	-	-	-
Usaramine	2647	-	-	-	-	-	-	-	-	-	-	-	-	-	-	-	-	-	-	29±2	1±1	5±1	27±3	15±2	81±1
Senecionine-like	2675	-	-	-	-	-	-	-	-	-	-	-	-	4±1	3±1	5±1	-	-	-	3±1	-	-	26±2	34±2	11±1
Senecionine-like	2684	-	-	-	-	-	-	-	-	-	-	-	-	-	-	-	-	-	-	3±1	-	-	2±1	2±1	-
Unknown *seco*-PA	2728	-	-	-	-	-	-	-	-	6±1	-	-	-	-	-	-	-	-	-	-	-	-	-	-	-
Unknown *seco*-PA	2815	-	-	-	-	-	-	-	-	61±2	-	-	-	-	-	-	-	-	-	-	-	-	-	-	-
Unknown *seco*-PA	2866	-	-	-	-	-	-	-	-	20±2	-	-	-	-	-	-	-	-	-	-	-	-	-	-	-
Unknown *seco*-PA	2907	-	-	-	-	-	-	-	-	2±1	-	-	-	-	-	-	-	-	-	-	-	-	-	-	-

### Pyrrolizidine Alkaloids and Predator Response

Across all adults whose larvae fed on different host plants and on different plant parts, the concentration of individual PAs in the moths had a significant positive relationship with release by *N*. *clavipes* (Table 4). For adults whose larvae fed on each species of *Crotalaria*, this pattern persisted, but for *C*. *vitellina* we found no significant relationship. For *C*. *paulina* and *C*. *spectabilis*, no statistical analyses were possible, since adults whose larvae fed on these two host plants had a high PA concentration regardless of plant part, and 98 to 100% of them were released.

**Table 4:** Logistic regression results for the *Nephila clavipes* response (predation or release) in relation to the concentration of pyrrolizidine alkaloids in adults of *Utetheisa ornatrix* fed as larvae on leaves or unripe seeds of eight host plants.

Host plant	Equation	χ^2^	P^a^
*C*. *micans*	Logit P_i_ = -2.655 + (1.641 X_i_)	29.279	< 0.001
*C*. *paulina*	Not calculated: all individuals were released	-	-
*C*. *vitellina*	Logit P_i_ = 3.696 - (0.020 X_i_)	0.006	0.940
*C*. *ochroleuca*	Logit P_i_ = -2.547 + (1.772 X_i_)	40.464	< 0.001
*C*. *juncea*	Logit P_i_ = 0.526 + (0.887 X_i_)	5.384	0.020
*C*. *spectabilis*	Not calculated: only 1 of 37 individuals was preyed	-	-
*C*. *incana*	Logit P_i_ = -1.586 + (1.040 X_i_)	21.594	< 0.001
*C*. *pallida*	Logit P_i_ = -0.646 + (1.779 X_i_)	13.585	< 0.001
All species pooled together	Logit P_i_ = -0.751 + (0.890 X_i_)	129.535	< 0.001

^a^The likelihood ratio test

**Fig 7:**
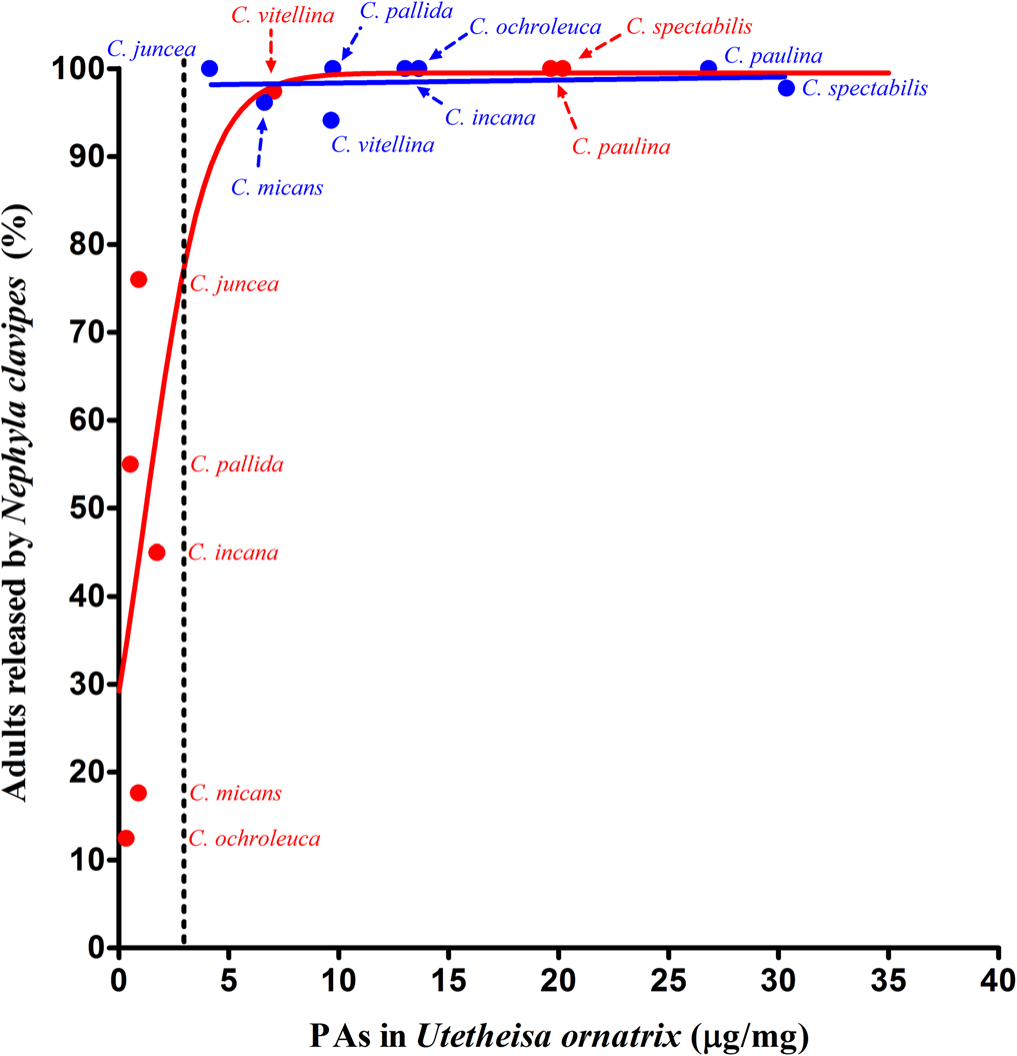
Non-linear relationship between the mean of pyrrolizidine alkaloid concentration (μg/mg dry weight) in adults of *Utetheisa ornatrix* fed on leaves of different *Crotalaria* species and the percentage of these adults released by *Nephila clavipes*. Red symbols stand for larvae fed on leaves and blue symbols stand for larvae fed on seeds. The dotted line is the concentration at which baits treated with monocrotaline and integerrimine in the *N-*oxide form (3.0 μg/mg) were 100% released by *Nephila clavipes*.

We found a significant positive relationship between the PA concentration of adults fed as larvae on leaves of different host plants and the percentage of adults released by *N*. *clavipes* [y = 99.5/(1+2.40e^-0.72x^), r^2^ = 0.83, Fig 7]. When larvae fed on host plants with a low PA concentration (*C*. *incana*, *C*. *micans*, *C*. *juncea*, *C*. *ochroleuca* and *C*. *pallida*), the percentage of release was low; conversely, when they fed on plants with a high PA concentration (*C*. *paulina*, *C*. *spectabilis* and *C*. *vitellina*), the percentage of release was high, nearly 100%.

### Predation Bioassay with Pure Pyrrolizidine Alkaloids

**Fig 8:**
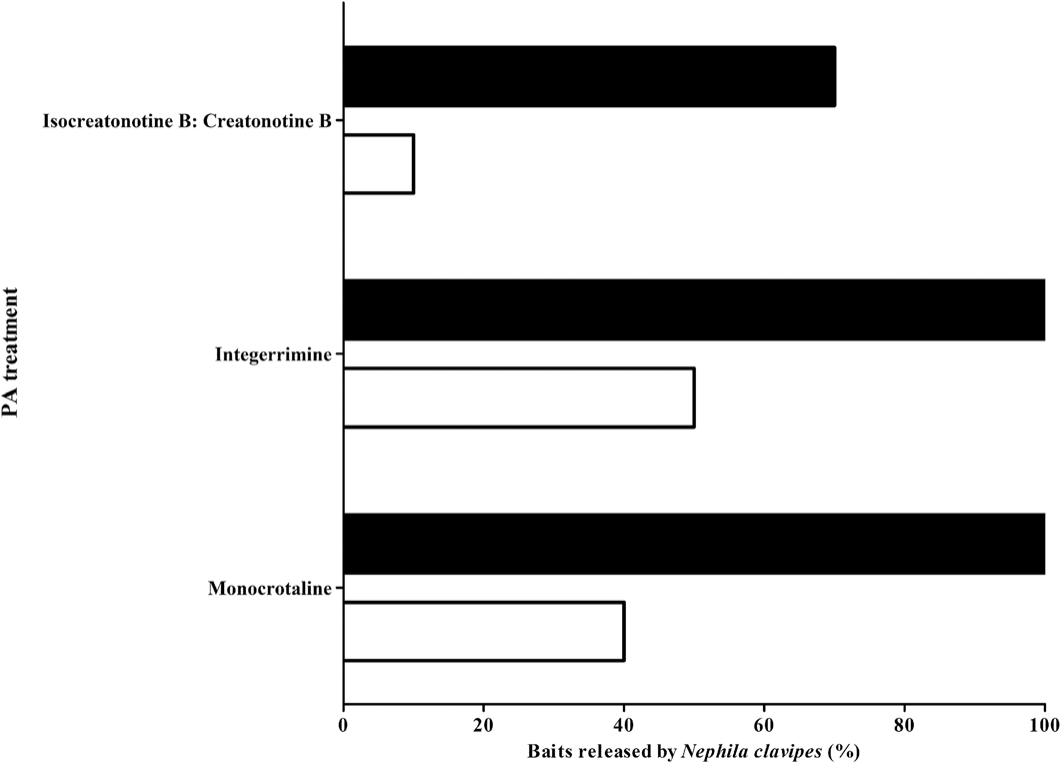
Percentage of baits treated with different PA *N*-oxides, which were released by the spider *Nephila clavipes*. In black, the concentration of PAs was 3.0 μg/mg; in white 0.3 μg/mg.

Baits treated with different *N*-oxide PA structures affected the percentages of baits (mealworm painted with PAs) released by *N*. *clavipes* (GLM binomial, χ^2^ = 9.692, P = 0.008). different PA concentrations also influenced the percentage of baits released by the spiders (χ^2^ = 27.442, df = 1, P < 0.001); while no interaction between factors was observed (χ^2^ = 0.008, df = 2, P = 0.996). *N*. *clavipes* released 100% of the baits treated with 3.0 **μ**g/mg *N*-oxides of monocrotaline or integerrimine, and 70% of the baits treated with the insect PA mixture creatonotine B: iso-creatotine B. For 0.3 **μ**g/mg, we found 60% release for monocrotaline, 50% for integerrimine, and 10% for the insect PAs (Fig 8). At the concentration of 5.0 **μ**g/mg, *N*. *clavipes* released 100% of baits treated with creatonotine B: iso-creatotine B.

### Host Plant Phylogenetic Hypothesis and Character Mapping

**Fig 9:**
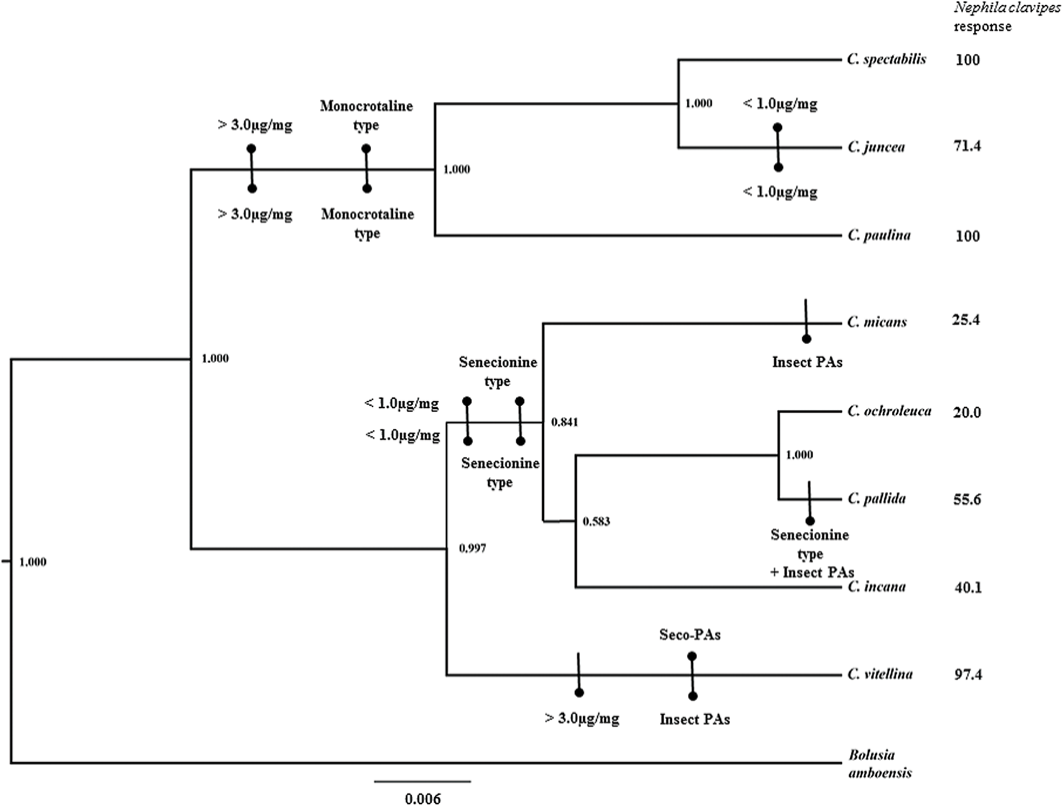
Bayesian inference topology for eight species of *Crotalaria* based on ITS sequences onto which are mapped the amount and type of PAs in leaves of the *Crotalaria* host plants (above) and in adults of *Utetheisa ornatrix* whose larvae fed on these plants (below). Response of the spider *Nephila clavipes* in relation to adults of *Utethesia ornatrix* is given beside each *Crotalaria* species. Numbers in each node indicate Bayesian posterior probabilities. The scale below the tree indicates the mean number of substitutions per site.

The eight species of *Crotalaria* assessed in this study were separated into three main clades, all highly supported by posterior probability values (Fig 9). Clade 1 included the species *C*. *paulina*, *C*. *spectabilis* and *C*. *juncea*, which showed monocrotaline-type PAs. High PA concentrations in leaves were found in *C*. *paulina* and *C*. *spectabilis*, but not in *C*. *juncea*. The moths feeding on leaves of plants of Clade 1 showed monocrotaline type PAs and a high PA concentration; most of these moths were released unharmed by the spiders. Clade 2 included the species *C*. *micans*, *C*. *ochroleuca*, *C*. *pallida* and *C*. *incana*, which contained senecionine-type PAs and small amounts of PA in the leaves. The moths reared on plants of this clade showed small PA amounts compared to those from plants of Clade 1, but a variation according to the class of PAs: adults reared on *C*. *micans* and *C*. *pallida* showed large amounts of insect PAs in the body, while the class senecionine was the main PA type found in adults reared on *C*. *ochroleuca* and *C*. *incana*; some of the adults were released by the spiders. Clade 3 included a single species, *C*. *vitellina*, which had seco-PAs and small amounts of PAs in the leaves. However, the PA content in moths reared on leaves of *C*. *vitellina* was high, with large amounts of insect PAs, and the spiders released almost all adults reared on this host species.

## Discussion

Our results indicated that PAs sequestered by *U*. *ornatrix* provide active chemical defense against predation, in a dose-dependent manner. The concentration of PAs in host plants where the larvae fed is a crucial factor for enhancing adult defense. Adults whose larvae fed on unripe seeds were almost 100% protected against *N*. *clavipes*, and showed high PA contents in their tissues. However, for those adults whose larvae fed on leaves, the protection against spider predation decreased nearly 40%; these adults, not coincidentally, showed low PA contents in their tissues. These results demonstrated that the high PA concentration in host plant seeds and low concentration in leaves might drive the spider response to *U*. *ornatrix*. Importantly, those adults whose larvae fed on leaves of *C*. *paulina*, *C*. *spectabilis* and *C*. *vitellina* had PA contents above 7.0 **μ**g/mg in their tissues, and were well protected against the spider. The high content of PAs in *C*. *paulina* and *C*. *spectabilis* leaves may explain this protective effect. However, this was not the case for *C*. *vitellina*; this species had seco-PAs as the main compounds, together with retronecine. *U*. *ornatrix* did not sequester the seco-PAs, but they showed a high content of insect PAs biosynthesized from retronecine, which maximized their chemical defense. Although it is well known that PAs are responsible for the chemical defense of *U*. *ornatrix* and other arctiine moths [5,14,17], a dose-dependent approach has been less thoroughly documented. For instance, Hristov and Conner [27] demonstrated that when larvae fed on *C*. *spectabilis* seeds, the adults were less palatable to bats compared to adults whose larvae fed on leaves. Ferro et al. [26] found similar results when bioassaying *U*. *ornatrix* reared on *C*. *pallida* against the spider *N*. *clavipes*. Additionally, Dussourd et al. [66] described the same pattern for *U*. *ornatrix* eggs: those with a high amount of monocrotaline were rejected by the predaceous coccinellid beetle *Coleomegilla maculata*, but those with a low content were preyed upon. Pure PAs topically applied in a dose-dependent manner on palatable baits and offered to *N*. *clavipes* showed similar results [11]; there was a positive relationship between PA concentration and the *N*. *clavipes* release response using pure PAs.

The dose-dependence response raised other questions: what is the amount needed to elicit the predator's release response, and is there a threshold of sequestration by the moth? The spiders usually released adults that contained over 3.0 **μ**g/mg PAs. However, adults whose larvae fed on *C*. *paulina* and *C*. *spectabilis* seeds showed ten times more PAs in their bodies. Feeding on high PA concentrations may not impose costs to *U*. *ornatrix* [47], and therefore larvae may prefer diets with a high PA content over diets with a low PA content [67]. However, larvae feeding on host plants with a very high PA concentration, such as in leaves and seeds of *C*. *paulina* and *C*. *spectabilis*, showed a sequestration threshold. Above 30 **μ**g/mg of PA concentration in their diet, the larvae were unable to sequester more PAs. Malcolm [68] found a similar pattern for the monarch butterfly *Danaus plexippus* fed as larvae on milkweed *Asclepias* host plants with different cardenolide levels. Monarchs sequester cardenolides from milkweed species with low cardenolide contents, but when the plant increases the cardenolide content more than three to fourfold, monarchs reach an upper asymptote for sequestration. The likelihood of an uptake threshold in *U*. *ornatrix*, to reduce the costs of sequestration and metabolism (e.g. *N*-oxidation), deserves further evaluation. Additionally, we know little about the threshold response of other potential predators of *U*. *ornatrix*. Our results showed that above 3.0 **μ**g/mg, adults were released by *N*. *clavipes*. However, the bat *Eptesicus fuscus* preyed on 70% of the adults of *U*. *ornatrix* from larvae fed on *C*. *spectabilis* leaves, and on 55% of the adults whose larvae fed on seeds [27]. Assuming that all *C*. *spectabilis* have a similar PA content, *E*. *fuscus* seems to tolerate a high concentration of PAs in relation to *N*. *clavipes*, which released all moths whose larvae fed on *C*. *spectabilis*. Therefore, sequestering a high amount of PAs may maximize the chemical defense against a broader predator spectrum. Furthermore, male moths biosynthesized the sex pheromone hydroxidanaidal from PAs [69] and passed on PAs to females as a nuptial gift; in turn, females endowed their eggs with this defense [66]. We suggest that the multiple uses of PAs by *U*. *ornatrix* may be responsible for the higher sequestration threshold.

To our knowledge, other pure defensive compounds sequestered from larval host plants by insects have not been tested in a dose-dependent manner. The closest example of a dose-dependent mechanism is found in the *Asplepias*-*Danaus* systems, where cardiac glycosides are sequestered from host plants of the subfamily Asclepiadoideae to protect *Danaus plexippus* against predation by birds [6]. Brower et al. [6] showed that the degree of unpalatability of the butterfly was linked to its host plants. However, there was a mismatch in the amount of cardenolides sequestered that caused unpalatability in *D*. *plexippus* and the cardenolide concentration in the host plants [68]. The low degree of unpalatability was attributed to the host plant with high cardenolide content, which was a non-native plant, and therefore they did not share a long evolutionary history [68].

The PA profile of adults of *U*. *ornatrix* varies in relation to the PA profiles of their larval host plant. The sequestration of unchanged PAs or transformation of plant PAs into insect PAs is also variable, as found for other arctiines [51,52,70,71]. Which PA is present in the adults seems to be unimportant for *U*. *ornatrix* chemical defense. Both *N*-oxides of monocrotaline and senecionine showed similar defensive efficiencies against *N*. *clavipes*. When the larvae feed on innocuous PAs such as retronecine, they are able to compensate for the structural inactivity by manufacturing their own PAs from plant precursors. These insect PAs exhibited efficacy comparable to that of monocrotaline- and senecionine-type PAs in protecting the moth against the spider. At high concentrations, analogous to those found in adults whose larvae fed on unripe seeds (3.0 **μ**g/mg), 80–100% of the baits treated with these alkaloids were released by the spider, irrespective of PA type. With a one-tenth concentration (0.3 **μ**g/mg), around 50% of the baits were released when the PAs were monocrotaline and senecionine, and 10% for the insect PAs tested. Siva and Trigo [11], using *N*. *clavipes* as the predator, found that a mixture of secionine:integerrimine *N*-oxide at 1.0 **μ**g/mg led to 100% release of baits treated with this mixture.

Insect PAs constitute an important defensive mechanism. These biosynthesized insect-specific PAs are produced by several arctiine moths, through the esterification of necine bases derived from plant PAs with necic acids of insect origin [16]. These alkaloids have never been reported for *U*. *ornatrix*, and we found them in adults fed as larvae on all eight *Crotalaria* species. Insect PAs are reported as precursors of PA-derived pheromones [6, 53], although both males and females showed these alkaloids. Their role as defensive compounds, however, has received little attention [e.g. 11]. Our study demonstrated that this kind of PA is important for chemical defense of *U*. *ornatrix*. For example, those adults fed as larvae on *C*. *vitellina* showed high insect PAs and low amounts of retronecine, and were efficiently protected against *N*. *clavipes*. An *N*-oxide mixture of creatonotine B:iso-creatonotine B, in a similar concentration to those found in *U*. *ornatrix* fed on *C*. *vitellina* and topically applied on a palatable prey, also showed 100% efficiency against *N*. *clavipes*. It is known that retronecine is not active against *N*. *clavipes* predation, but insect PAs are [11]. Therefore, the transformation of necine bases into insect PAs may have been selected under predation pressure. Similarly to other PA compounds sequestered from plants, these insect-PAs have a dual role: chemical protection against predators, and precursors of sex pheromones in males of specialist lepidopterans.

Finally, the sequestration of defensive PAs by *U*. *ornatrix* from *Crotalaria* species is linked with *Crotalaria* phylogeny to some extent. Our phylogenetic analysis with the eight *Crotalaria* species showed one clade with high leaf PA content and another clade with low leaf PA content; both clades showed high PA contents in seeds. *Crotalaria* phylogeny may affect the chemical defense of *U*. *ornatrix*, when larvae feed on leaves, but not on seeds. Therefore, we can expect that if all other traits are equal, larvae would feed on both leaves and seeds of plants from clades with high PA content in the leaves, and would feed only on seeds in species from the clade with low leaf PA concentration. The extent to which the selective pressure of *U*. *ornatrix* on *Crotalaria* species drove the differences in PA concentration and profile among the *Crotalaria* species remains to be determined. For example, it is unknown if the shared evolutionary history between *Crotalaria* and *U*. *ornatrix* could have coevolved, affecting the chemical defenses in both the plant and the moth. On the other hand, when *U*. *ornatrix* or its ancestral line began to use *Crotalaria* as host plants, the patterns of plant PA defense could have already been established due to other selective forces, e.g. other herbivores.

In conclusion, the main factor in the chemical defense of *U*. *ornatrix* is the amount of PA sequestered or transformed from their host plants. Feeding on plants or parts of plants with high PA contents enhances the protection of *U*. *ornatrix* against predators. The presence of a non-active PA, such as retronecine, may not constitute a constraint on *U*. *ornatrix* chemical defense. The moth overcomes these limitations by maximizing the production of insect PAs, which have an antipredator role. Another constraint could be non-native *Crotalaria* host plants that impair both larval and adult performance, increasing the development time and decreasing the pupal weight ([21], J.R. Trigo, personal observation). However, this impairment may not affect the chemical defense of adults when the plants contain enough PAs to be sequestered by larvae. We suggest that the high degree of specialization of *U*. *ornatrix* on PAs in *Crotalaria* led to an efficient uptake of these compounds, independently of other nutritional or toxic constraints on its larval diet.
